# Molecular Weight Distribution of Branched Polymers: Comparison between Monte Carlo Simulation and Flory-Stockmayer Theory

**DOI:** 10.3390/polym15071791

**Published:** 2023-04-04

**Authors:** Chengyuan Wen, Roy Odle, Shengfeng Cheng

**Affiliations:** 1 Key Laboratory of Oceanographic Big Data Mining and Application of Zhejiang Province, School of Information Engineering, Zhejiang Ocean University, Zhoushan 316022, China; 2 Department of Physics, Center for Soft Matter and Biological Physics, and Macromolecules Innovation Institute, Virginia Tech, Blacksburg, VA 24061, USA; 3 SABIC, 1 Lexan Lane, Mt. Vernon, IN 47620, USA; 4 Department of Mechanical Engineering, Virginia Tech, Blacksburg, VA 24061, USA

**Keywords:** branched polymer, Monte Carlo simulation, Flory-Stockmayer theory, molecular weight distribution, polyetherimide, step-growth polymerization

## Abstract

It is challenging to predict the molecular weight distribution (MWD) for a polymer with a branched architecture, though such information will significantly benefit the design and development of branched polymers with desired properties and functions. A Monte Carlo (MC) simulation method based on the Gillespie algorithm is developed to quickly compute the MWD of branched polymers formed through step-growth polymerization, with a branched polyetherimide from two backbone monomers (4,4${}^{\prime }$-bisphenol A dianhydride and m-phenylenediamine), a chain terminator (phthalic anhydride), and a branching agent (tris[4-(4-aminophenoxy)phenyl] ethane) as an example. This polymerization involves four reactions that can be all reduced to a condensation reaction between an amine group and a carboxylic anhydride group. A comparison between the MC simulation results and the predictions of the Flory-Stockmayer theory on MWD shows that the rates of the reactions are determined by the concentrations of the functional groups on the monomers involved in each reaction. It further shows that the Flory-Stockmayer theory predicts MWD well for systems below the gel point but starts to fail for systems around or above the gel point. However, for all the systems, the MC method can be used to reliably predict MWD no matter if they are below or above the gel point. Even for a macroscopic system, a converging distribution can be quickly obtained through MC simulations on a system of only a few hundred to a few thousand monomers that have the same molar ratios as in the macroscopic system.

## 1. Introduction

The molecular weight distribution (MWD) and architecture are two important characteristics of a polymer [1]. They strongly affect material properties such as dynamic moduli, fracture toughness, glass transition temperature, and viscosity [1,2,3]. Experimental methods for an accurate determination of MWD are thus of great interest [3,4,5]. Theoretically, it is also highly desirable if the MWD of a polymer can be predicted a priori based on the knowledge of the polymerization reaction involved without even synthesizing the polymer. Such a theoretical method will be a valuable tool that is not only useful for understanding experimental measurements, but also beneficial for other theories and models aiming to predict polymer properties. For example, Nichetti and Manas-Zloczower [6] proposed a theoretical model to predict the viscosity of a polymer melt based on its MWD that was determined by fitting the data from gel permeation chromatography to statistical distribution functions. A method to quickly generate its MWD without the necessity of experimental input thus can advance the predictive capability of such theories [7].

A theory on the constitution and molecular size distribution of a step-growth polymer was proposed by Flory and Stockmayer several decades ago [8,9,10,11,12,13], and has been frequently used to determine the gel point. Flory studied the polymerization of bifunctional monomers mixed with trifunctional and tetrafunctional branching units, and made two fundamental assumptions [8,9,10]. First, the same functional group has the same probability to react with another group, and this probability is not affected by the length of the polymer to which the functional group belongs, as well as the position of the functional group on that polymer. Secondly, ring polymers are not formed. Stockmayer extended the theory to branching units with arbitrary functionalities and derived the Stockmayer formula for the number of polymer chains with a given composition, though ring structures were still excluded [11,12,13]. The predictions of the Flory-Stockmayer theory [8,9,10,11,12,13], including the gel point and the average molecular weight, have been tested experimentally [14,15,16,17,18,19,20]. However, the entire MWD is hard to probe experimentally, and often only the average molecular weight is measured. Practically, it is also difficult to directly predict MWD using the Flory-Stockmayer theory because of the mathematical complexity involved in computing the amount of all possible molecules in a branched polymer system. Furthermore, the Flory-Stockmayer theory is expected to be only valid below the gel point. Schamboeck et al. [21] suggested a new theoretical method based on directed random graphs to overcome the complexity of the Flory-Stockmayer theory, but the distribution beyond the gel point was not presented. Beyond the gel point, the formation of cyclics and closed loops in a branched structure becomes significant, and theories ignoring their presence may fail [22,23].

Many computational methods, such as molecular dynamics simulations and dissipative particle dynamics based on either all-atom [24] or coarse-grained models [25,26,27], can be used for simulating chemical reactions including polymerization. However, it can be challenging for these methods to treat a nearly fully reacted system because of the limitation on their accessible time scales. Furthermore, the formation of branched polymers is even more difficult for these methods to handle, as when the extent of the reaction grows larger, it becomes harder to visit monomers already incorporated into a polymer, though they may still possess active functional groups. On the other hand, a polymerization process is much more amenable to Monte Carlo (MC) simulations [28,29,30].

MC methods are a class of techniques based on random sampling to numerically solve problems that have a probabilistic interpretation [31]. MC methods have broad applications in polymer science [32,33,34], especially in polymer reaction engineering [28,33]. Johnson and O’Driscoll [35] used an MC simulation to study sequence distributions in step-growth copolymerization. Tobita applied MC simulation to a wide range of polymerization problems, including free-radical polymerization [36,37,38], emulsion polymerization [39,40,41], long-chain branching and random scission [42,43], and living radical polymerization [44,45]. Hadicke and Stutz [46] used an amine-cured epoxy as an example to compare the structure of step-growth networks obtained via MC simulation to that predicted by the branching theory. He et al. [47,48] applied an MC method to simulate self-condensing vinyl polymerization in the presence of multifunctional initiators, and probed the role of reactive rate constants. Rouault and Milchev [49], and He et al. [50] performed MC simulations to study the kinetics and chain length distributions in living polymerization. Prescott [51] used an MC model to show that chain-length-dependent termination plays a significant role in living/controlled free-radical polymerization systems containing reversible transfer agents. In a series of papers, Al-Harthi et al. [52,53,54,55] used dynamic MC simulations to study atom transfer radical polymerization. Polanowski et al. [56,57] and Bannister et al. [58] used MC methods to study branching and gelation in the living copolymerization of vinyl and divinyl monomers. Recently, Polanowski et al. [59] used MC simulations to investigate the polymerization of star and dendritic polymers. Lyu et al. [22,23] used a similar model to study the atom transfer radical polymerization and the conventional free-radical polymerization of divinyl monomers, and checked the applicability of the Flory-Stockmayer theory in such systems. Gao et al. [60,61] used kinetic MC methods to simulate free-radical copolymerization processes, and discussed how to accelerate such simulations using scaling approaches. Meimaroglou et al. [62] proposed an MC algorithm to calculate the MWD for linear polymers, and the bivariate molecular weight - long chain branching distribution for highly branched polymers. They also used MC simulation to investigate the molecular, topological, and solution properties of highly branched low-density polyethylene [63], and the ring-opening homopolymerization of L,L-Lactide [64]. Jin et al. [65] employed kinetic MC simulations to study the cross-linked network formation between hydroxyl-terminated poly(dimethylsiloxane) and triisocyanate. Iedema et al. [66] developed an MC simulation model including both branching and random scission to calculate the molecular weight and branching distributions, and compared their calculations to experimental measurements on high-density polyethylene. Yaghini and Iedema [67] compared the results on low-density polyethylene from such MC simulations to the predictions of a multiradical model based on a Galerkin finite element approach. De Keer et al. [68] used a matrix-based kinetic MC framework to reveal the effects of intramolecular reactions on the variation of the chain length distribution and its averages for a network polymer formed via a step-growth mechanism, with the intention to go beyond the equation derived by Carothers [69], Flory [8,70,71], and Stockmayer [11,12,13].

One important application of MC simulations is to quickly compute MWD [72,73,74,75,76]. In MC simulations, all structures, including rings and networks allowed by a polymerization reaction, can be produced [77], no matter whether the system is below or beyond the gel point [78]. Various schemes can also be implemented to capture different polymerization kinetics, which thus allows us to test the specific assumptions made by a theory. The Gillespie algorithm can be used to speed up the kinetics of a reaction in silico and enable a reactive system to quickly reach a steady state [79,80]. In this paper, we develop an MC simulation model based on the Gillespie algorithm to study the formation of branched chains via step-growth polymerization. This model is applied to the polymerization of polyetherimides (PEIs) in the presence of chain terminators and branching agents. The results from the MC simulations are used to test the Flory-Stockmayer theory [8,9,10,11,12,13], including its assumption on the reaction rates.

This paper is organized as follows. In Section 2, the Flory-Stockmayer theory is introduced, the technical challenge of computing MWD with this theory is discussed, and an approximation method is proposed. In Section 3, the MC model of the polymerization process of the branched PEIs being studied is described in detail. This particular polymerization system serves as an example to facilitate understanding. However, the MC model described can be applied to other branched polymers as well. In Section 4, the MC results are compared to the predictions of the Flory-Stockmayer theory. Practically, computations of MWD can only be executed for a small system either with the Flory-Stockmayer theory or the MC model. Therefore, a discussion on the effect of finite system size is included. Although the emphasis is on stoichiometric, fully reacted systems, those that are only partially reacted and/or nonstoichiometric are also discussed in Section 4. Finally, a brief summary is provided in Section 5.

## 2. The Flory-Stockmayer Theory of Step-Growth Polymers

Flory and Stockmayer considered a general step-growth polymer that consists of two types of monomers, *A* and *B* [8,9,10,11,12,13]. All reactions occur between *A* and *B*. There are *i* type-*A* monomers, denoted as $A_{1}$, $A_{2}$, and …, $A_{i}$. To simplify the notation, $A_{q}$ with q∈{1,2,…,i} is also used to denote the number of $A_{q}$ monomers. Similarly, there are *j* type-*B* monomers, and the corresponding numbers are $B_{1}$, $B_{2}$, …, $B_{j}$, respectively. The symbol $f_{q}$ denotes the functionality of an $A_{q}$ monomer, where q∈{1,2,…,i}, i.e., there are $f_{q}$ functional groups on an $A_{q}$ monomer, each of which can form a bond with another functional group on a $B_{h}$ monomer, where h∈{1,2,…,j}. The functionality of a $B_{h}$ monomer is denoted as $g_{h}$. The Flory-Stockmayer theory can be applied to a polymerized state, where a fraction ($p_{A}$) of all the functional groups on the type-*A* monomers have reacted with a fraction ($p_{B}$) of all the functional groups on the type-*B* monomers. Therefore,

(1) $$p_{A}\sum_{q=1}^{i}f_{q}A_{q}=p_{B}\sum_{h=1}^{j}g_{h}B_{h}$$

In this paper, the systems with $\sum_{q=1}^{i}f_{q}A_{q}=\sum_{h=1}^{j}g_{h}B_{h}$, and thus, $p_{A}=p_{B}$ are called stoichiometric systems, while those with $\sum_{q=1}^{i}f_{q}A_{q}\neq \sum_{h=1}^{j}g_{h}B_{h}$ and $p_{A}\neq p_{B}$ are called nonstoichiometric. The systems with $p_{A}$ or $p_{B}$, or both, equal to 1, are fully reacted.

N{m,n} denotes the number of molecules formed by $m_{q}$ monomers of sub-type $A_{q}$ and $n_{h}$ monomers of sub-type $B_{h}$, with *q* running from 1 to *i* and *h* running from 1 to *j*. Here, {m,n} is a shorthand of $\left\{m_{1},m_{2}.,\ldots ,m_{i},n_{1},n_{2},\ldots ,n_{j}\right\}$, which denotes the monomer composition of a given molecule. The Flory-Stockmayer theory predicts that

(2) $$\begin{array}{ccc} N\{m,n\} & = & K\frac{\left(\sum_{q=1}^{i}f_{q}m_{q}-\sum_{q=1}^{i}m_{q}\right)!}{\left(\sum_{q=1}^{i}f_{q}m_{q}-\sum_{q=1}^{i}m_{q}-\sum_{h=1}^{j}n_{h}+1\right)!}\\ & & \cdot \frac{\left(\sum_{h=1}^{j}g_{h}n_{h}-\sum_{h=1}^{j}n_{h}\right)!}{\left(\sum_{h=1}^{j}g_{h}n_{h}-\sum_{h=1}^{j}n_{h}-\sum_{q=1}^{i}m_{q}+1\right)!}\\ & & \cdot \prod_{q=1}^{i}\frac{x_{q}^{m_{q}}}{m_{q}!}\prod_{h=1}^{j}\frac{y_{h}^{n_{h}}}{n_{h}!} \end{array}$$

where

(3) $$\begin{array}{ccc} x_{q} & = & \frac{f_{q}A_{q}}{\sum_{l=1}^{i}f_{l}A_{l}}\frac{p_{B}{\left(1-p_{A}\right)}^{f_{q}-1}}{\left(1-p_{B}\right)} \end{array}$$

(4) $$\begin{array}{ccc} y_{h} & = & \frac{g_{h}B_{h}}{\sum_{l=1}^{j}g_{l}B_{l}}\frac{p_{A}{\left(1-p_{B}\right)}^{g_{h}-1}}{1-p_{A}} \end{array}$$

(5) $$\begin{array}{ccc} K & = & \frac{\left(1-p_{A}\right)\left(1-p_{B}\right)}{p_{B}}\sum_{q=1}^{i}f_{q}A_{q}=\frac{\left(1-p_{A}\right)\left(1-p_{B}\right)}{p_{A}}\sum_{h=1}^{j}g_{h}B_{h} \end{array}$$

Equation (2) is called the Stockmayer formula, which gives the number of molecules of any monomer compositions. However, it is practically difficult to compute MWD from the Stockmayer formula, as all the possible combinations in $\left\{m_{1},m_{2}.,\ldots ,m_{i},n_{1},n_{2},\ldots ,n_{j}\right\}$ have to be taken into account. Since $m_{q}$ runs from 1 to $A_{q}$ for q∈{1,2,…,i}, and $n_{h}$ runs from 1 to $B_{h}$ for h∈{1,2,…,j}, the total number of possible molecules is $\prod_{q=1}^{i}A_{q}!\times \prod_{h=1}^{j}B_{h}!$. This number is huge when there are many sub-types (i.e., large *i* and *j*) and/or large numbers (i.e., large $A_{q}$ and $B_{h}$) of monomers involved in a polymerization process.

For a molecule with composition {m,n}, the total number of monomers is $\sum_{q=1}^{i}m_{q}+\sum_{h=1}^{j}n_{h}$. Since the Flory-Stockmayer theory does not consider rings, the total number of bonds in this molecule must be $\sum_{q=1}^{i}m_{q}+\sum_{h=1}^{j}n_{h}-1$. When $p_{A}=p_{B}=1$, all the functional groups have reacted, and in a given molecule, the total number of the functional groups on all the type-*A* monomers is equal to the total number of the functional groups on all the type-*B* monomers. This number must also be equal to the total number of bonds in that molecule. Namely, for $p_{A}=p_{B}=1$ there are two identities,

(6) $$\sum_{q=1}^{i}f_{q}m_{q}=\sum_{q=1}^{i}m_{q}+\sum_{h=1}^{j}n_{h}-1$$

and

(7) $$\sum_{h=1}^{j}g_{h}n_{h}=\sum_{h=1}^{j}n_{h}+\sum_{q=1}^{i}m_{q}-1$$

These two identities can help us simplify the Stockmayer formula for stoichiometric, fully reacted systems. Note that in Equation (2), the terms involving $\left(1-p_{A}\right)$ and $\left(1-p_{B}\right)$ appear as

$${\left(1-p_{A}\right)}^{\sum_{q=1}^{i}f_{q}m_{q}-\sum_{q=1}^{i}m_{q}-\sum_{h=1}^{j}n_{h}+1}$$

and

$${\left(1-p_{B}\right)}^{\sum_{h=1}^{j}g_{h}n_{h}-\sum_{h=1}^{j}n_{h}-\sum_{q=1}^{i}m_{q}+1}$$

These terms can be dropped out because of Equations (6) and (7). As a result, for fully reacted stoichiometric systems with $p_{A}=p_{B}=1$, the Stockmayer formula is simplified as

(8) $$N\left\{m,n\right\}=K\left(\sum_{q=1}^{i}f_{q}m_{q}-\sum_{q=1}^{i}m_{q}\right)!\left(\sum_{h=1}^{j}g_{h}n_{h}-\sum_{h=1}^{j}n_{h}\right)!\prod_{q=1}^{i}\frac{x_{q}^{m_{q}}}{m_{q}!}\prod_{h=1}^{j}\frac{y_{h}^{n_{h}}}{n_{h}!}$$

with

(9) $$\begin{array}{ccc} x_{q} & = & \frac{f_{q}A_{q}}{\sum_{l=1}^{i}f_{l}A_{l}} \end{array}$$

(10) $$\begin{array}{ccc} y_{h} & = & \frac{g_{h}B_{h}}{\sum_{l=1}^{j}g_{l}B_{l}} \end{array}$$

(11) $$\begin{array}{ccc} K & = & \sum_{q=1}^{i}f_{q}A_{q}=\sum_{h=1}^{j}g_{h}B_{h} \end{array}$$

Computing N{m,n} is not easy, as it contains many factorials. The calculation can be expedited using the Stirling approximation,

(12) $$\log n!\approx \log\left(\sqrt{2\pi n}\right)+n\log\left(\frac{n}{e}\right)+\log\left(1+\frac{1}{12n}\right)$$

Then, for fully reacted stoichiometric systems, the Stockmayer formula can be approximated logarithmically as

(13) $$\begin{array}{ccc} \log N\{m,n\} & \approx & \log K+\log\left(\sum_{q=1}^{i}f_{q}m_{q}-\sum_{q=1}^{i}m_{q}\right)!+\log\left(\sum_{h=1}^{j}g_{h}n_{h}-\sum_{h=1}^{j}n_{h}\right)!\\ & & +\sum_{q=1}^{i}\left(m_{q}\log x_{q}-\log m_{q}!\right)+\sum_{h=1}^{j}\left(n_{h}\log y_{h}-\log n_{h}!\right) \end{array}$$

The computation of MWD from N{m,n} can be further accelerated by noting that not all the combinations in {m,n} will yield a molecule. For a fully reacted stoichiometric system where $p_{A}=p_{B}=1$, Equations (1), (6), and (7) can be used to reduce the total number of {m,n}. For the branched PEIs considered in this paper (see Section 3), $f_{1}=1$, $f_{2}=2$, $g_{1}=2$, and $g_{2}=3$. The constraints become

(14) $$m_{1}+2m_{2}=2n_{1}+3n_{2}$$

and

(15) $$m_{2}=n_{1}+n_{2}-1$$

Equations (14) and (15) combined yield

(16) $$m_{1}=n_{2}+2$$

Equations (15) and (16) indicate that $m_{1}$ and $m_{2}$ are totally constrained by $n_{1}$ and $n_{2}$ in an allowed composition. Furthermore, since $m_{2}$ must be nonnegative, $n_{1}$ and $n_{2}$ cannot be zero at the same time. The time complexity to enumerate all possible molecules is thus $O(B_{1}B_{2})$, which is approximately $O(Z^{2})$, with *Z* being the system size (i.e., the total number of monomers prior to polymerization). This time complexity is acceptable for small systems. However, if there are multiple sub-types of monomers, then the time complexity will increase exponentially as $O(Z^{w})$, where *w* is the number of monomer sub-types. For partially reacted or nonstoichiometric systems where $p_{A}$ or $p_{B}$ are less than 1, the constraints that help reduce the number of possible {m,n} are lost. Then, computing MWD from N{m,n} has to rely on Equation (2), and this will become more challenging, even though the Stirling approximation may still be used. In these situations, the MC model described below will serve as a solution as it does not suffer from such limitations and the time complexity of computing MWD with MC simulations is always O(Z)×k, where *k* is the number of MC runs needed to obtain the desired statistics. Usually, *k* is about $10^{3}$ to $10^{4}$.

## 3. Monte Carlo Model of Polymerization of Branched Polyetherimides (PEIs)

**Figure 1:**
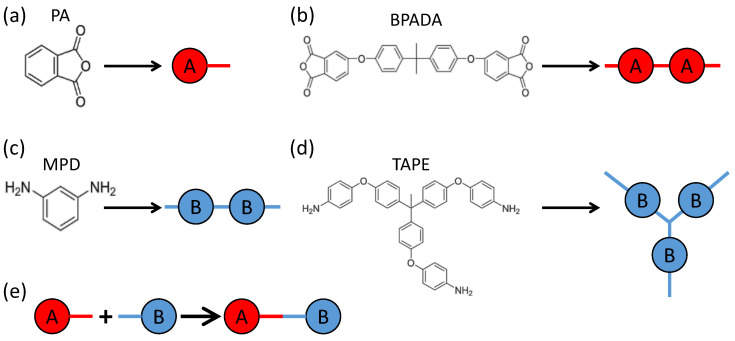
(**a**–**d**): The representation of the four types of monomers of the branched PEIs in the MC simulation model. Each functional group containing one amine is mapped to a *B* bead. Each functional group containing one carboxylic anhydride is mapped to an *A* bead. (**e**): Each *A* bead can form a bond with a *B* bead, mimicking the condensation reaction between an amine group and a carboxylic anhydride group in the polymerization of PEIs.

Four types of monomers are involved in the formation of the branched PEIs, including 4,4′-bisphenol A dianhydride (BPADA), m-phenylenediamine (MPD), phthalic anhydride (PA), and tris[4-(4-aminophenoxy)phenyl] ethane (TAPE) [81]. The chemical structures of these monomers are shown in Figure 1. The involved reaction is the condensation reaction between an amine group on MPD or TAPE, and a carboxylic anhydride group on BPADA or PA. In the notation of the Flory-Stockmayer theory, PA is monomer $A_{1}$ with $f_{1}=1$, BPADA is monomer $A_{2}$ with $f_{2}=2$, MPD is monomer $B_{1}$ with $g_{1}=2$, and TAPE is monomer $B_{2}$ with $g_{2}=3$. Out of these monomers, PA is an end capper to terminate a chain, and TAPE is a trifunctional branching agent. Figure 1 shows the representation of these monomers in our MC model. Each functional group containing one carboxylic anhydride is mapped to an *A* bead, and that containing one amine is mapped to a *B* bead. Each *A* bead can react with a *B* bead to form a bond (i.e., A+B→AB), which describes the condensation reaction between an amine group and a carboxylic anhydride group.

**Figure 2:**
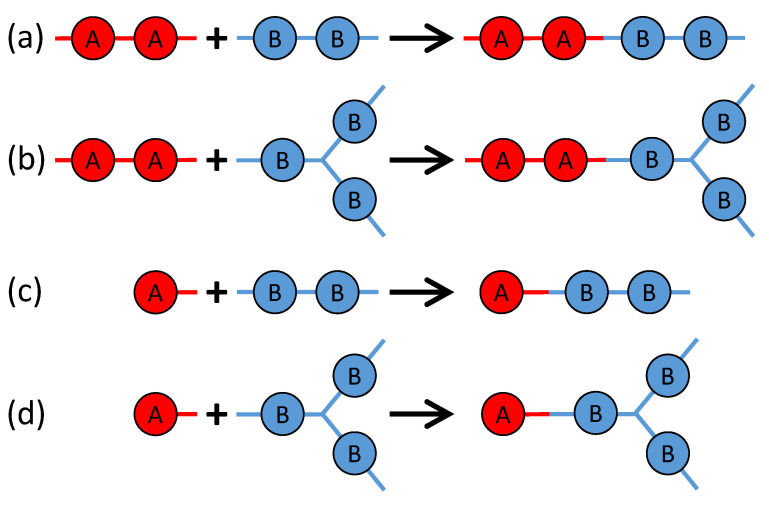
The four reactions occurring in the polymerization of the branched PEIs: (**a**) Reaction 1: BPADA + MPD, (**b**) Reaction 2: BPADA + TAPE, (**c**) Reaction 3: PA + MPD, and (**d**) Reaction 4: PA + TAPE.

There are four possible reactions among the above four types of monomers, as sketched in Figure 2. Reaction 1 is between BPADA and MPD, which leads to the formation of a PEI backbone. Reaction 2 is between BPADA and TAPE, which results in branching. Reaction 3 is between PA and MPD, which terminates a PEI chain. Reaction 4 is between PA and TAPE, which consumes one amine group on TAPE and effectively reduces its functionality by 1.

With the mapping in Figure 1 and the reaction scheme in Figure 2, MC simulations are performed to study the branched PEI polymerization. The Gillespie algorithm is adopted to speed up MC simulations. Since only the final chain structures are concerned, the random process in the typical Gillespie algorithm, which determines the time interval after which the next reaction occurs, is neglected. At each MC step, all four reactions will have a probability to occur, and the reaction rate of a particular reaction is determined by a rate constant and the concentration of the unreacted functional groups on the two types of monomers involved in that reaction. Mathematically, the probability of reaction *l* is proportional to

(17) $$P_{l}\left(L_{l}+R_{l}\to L_{l}R_{l}\right)=k_{l}n_{L_{l}}n_{R_{l}}$$

where $L_{l}$ ($R_{l}$) represents the reactant consisting of *A* (*B*) beads, $k_{l}$ is a rate constant, $n_{L_{l}}$ ($n_{R_{l}}$) is a quantity that depends on the concentration of the reactant $L_{l}$ ($R_{l}$), and l∈{1,2,3,4} indexes the possible reactions sketched in Figure 2. Specifically, $L_{1}$ and $L_{2}$ are BPADA, $L_{3}$ and $L_{4}$ are PA, $R_{1}$ and $R_{3}$ are MPD, and $R_{2}$ and $R_{4}$ are TAPE. Since all the four reactions can be reduced to the reaction between an *A* bead and a *B* bead (i.e., the reaction between a functional group containing one amine and another functional group containing one carboxylic anhydride), as shown in Figure 1e, $k_{l}$ will be set as a constant *k* for all the four reactions, and $n_{L_{l}}$ and $n_{R_{l}}$ can be expressed as

(18) $$\begin{array}{ccc} & & n_{L_{1}}=n_{L_{2}}=n_{\mathrm{BPADA}}^{A}\\ & & n_{L_{3}}=n_{L_{4}}=n_{\mathrm{PA}}^{A}\\ & & n_{R_{1}}=n_{R_{3}}=n_{\mathrm{MPD}}^{B}\\ & & n_{R_{2}}=n_{R_{4}}=n_{\mathrm{TAPE}}^{B} \end{array}$$

where $n_{\mathrm{BPADA}}^{A}$, $n_{\mathrm{PA}}^{A}$, $n_{\mathrm{MPD}}^{B}$, and $n_{\mathrm{TAPE}}^{B}$ are the concentrations of active functional groups on each type of monomers. In other words, $n_{L_{l}}$ ($n_{R_{l}}$) is the concentration in terms of the number of unreacted *A* (*B*) beads on the reactant $L_{l}$ ($R_{l}$). The particular reason of writing $n_{L_{l}}$ and $n_{R_{l}}$ in this way will be discussed in Section 4.1.

**Figure 3:**
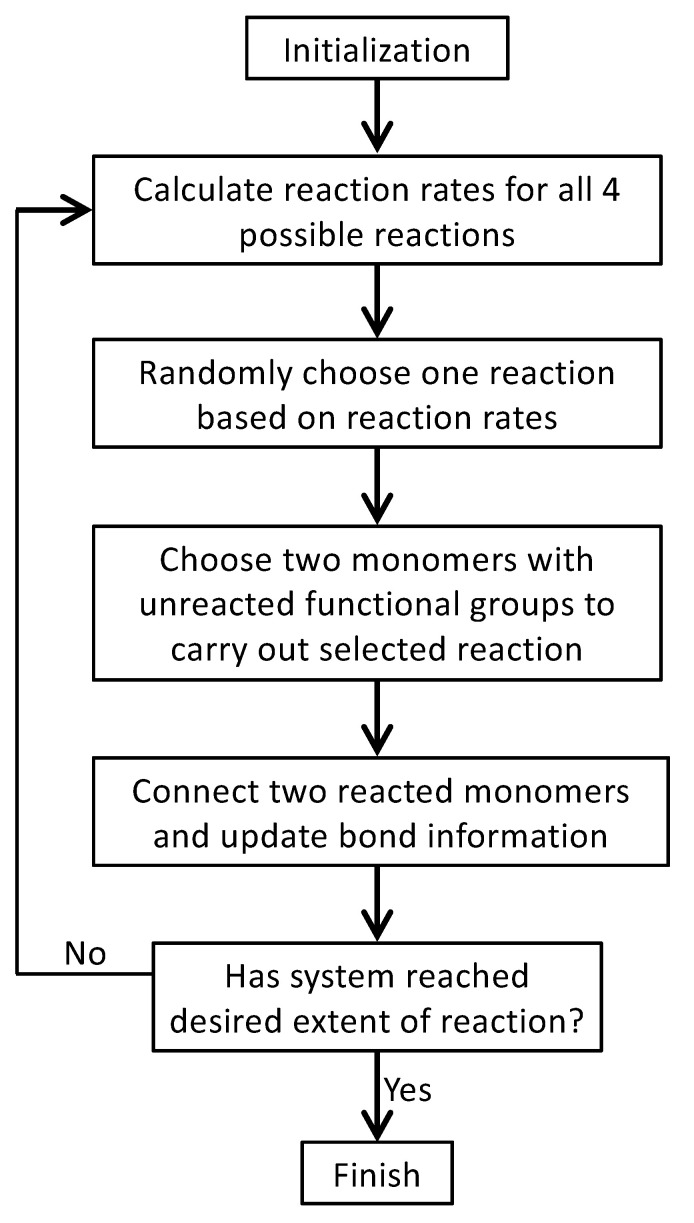
The flow chart of the MC simulation model of the branched PEI polymerization.

At each MC step, the probability of Reaction *l* to be chosen is equal to $P_{l}/\sum_{q=1}^{4}P_{q}$. After a reaction is selected, a pair of $L_{l}$ and $R_{l}$ that have unreacted functional groups (i.e., with unreacted *A* and *B* beads, respectively) is randomly chosen to react. Then, the system status is updated, including the bond information between the monomers and the identity of monomers with unreacted functional groups. The MC process is repeated for the updated system until no more reactions can occur or when the system has reached a desired extent of reaction. The flow chart of the MC simulation model is shown in Figure 3. Note that in this model, backward reactions are not allowed, which means that once formed, the bond between an *A* bead and a *B* bead cannot break. However, the model permits the formation of rings, loops, and networks.

## 4. Results and Discussion

### 4.1. Rate Constant k

Equation (18) indicates that the reaction rate $P_{l}$ is based on the concentrations of the unreacted functional groups (i.e., unreacted *A* beads or *B* beads) on the reactants involved in that reaction. However, $P_{l}$ can also be computed from the concentrations of the available reactants themselves, i.e., the monomer concentrations. In this case, the reaction rate $P_{l}$ can be written in the same way as in Equation (17), but with Equation (18) replaced by

(19) $$\begin{array}{ccc} & & n_{L_{1}}=n_{L_{2}}=n_{\mathrm{BPADA}}\\ & & n_{L_{3}}=n_{L_{4}}=n_{\mathrm{PA}}\\ & & n_{R_{1}}=n_{R_{3}}=n_{\mathrm{MPD}}\\ & & n_{R_{2}}=n_{R_{4}}=n_{\mathrm{TAPE}} \end{array}$$

where $n_{\mathrm{BPADA}}$, $n_{\mathrm{PA}}$, $n_{\mathrm{MPD}}$, and $n_{\mathrm{TAPE}}$ are the concentrations of monomers available for reactions (i.e., monomers with at least one unreacted functional group). Taking into account the functionality of monomers, it is noted that $n_{\mathrm{PA}}^{A}=n_{\mathrm{PA}}$, while $n_{\mathrm{BPADA}}^{A}\leq 2n_{\mathrm{BPADA}}$, $n_{\mathrm{MPD}}^{A}\leq 2n_{\mathrm{MPD}}$, and $n_{\mathrm{TAPE}}^{A}\leq 3n_{\mathrm{TAPE}}$. For the last three relations, the equality only holds at the first reaction.

To check which way of computing the reaction rates yields results that are more applicable to realistic systems, a test is performed with a simple system consisting of only PA and TAPE monomers, as shown in Table 1. For this system, there are only four possible final products, including single TAPEs and TAPEs connected with one, two, or three PAs, respectively.

**Figure 4:**
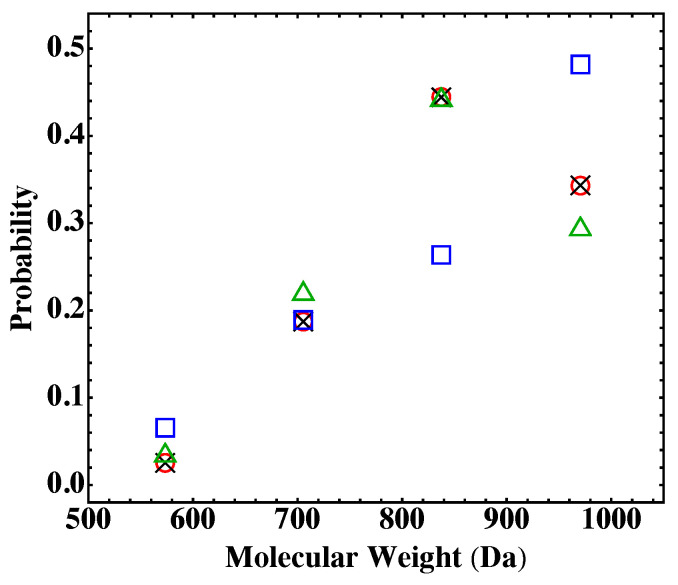
Probabilities of the four possible final products for the system in Table 1. The results are from the Flory-Stockmayer theory (red circles), the MC simulations using Equation (18) (black crosses), the MC simulations using Equation (19) (blue squares), and a simple statistical model discussed in the main text (green triangles). The MC results are the averages of 10,000 runs.

Figure 4 shows the results on the probability distribution of the four final products for the system in Table 1, for which gelation is not a concern. The comparison shows that the results from the MC simulations based on Equation (18) agree with the Flory-Stockmayer theory, while those based on Equation (19) do not. It indicates that Equation (18) is consistent with the assumption made by the Flory-Stockmayer theory, i.e., all the active functional groups have the same probability to react with another group. Therefore, a monomer with more unreacted functional groups has a larger probability to participate in a reaction than another monomer of the same type but with fewer active functional groups. However, Equation (19) assumes that all the monomers of the same type have the same probability to form a bond with another monomer. In this scheme, the same type of monomers having different numbers of unreacted functional groups, as long as they are not 0, have the same probability to react. This scheme effectively suppresses the reactivity of the monomers possessing more active functional groups. Since the Flory-Stockmayer theory has been validated experimentally for systems below the gel point [14,15,16,17,18,19,20], we conclude that the reaction rates based on Equation (18) should be used in the MC simulations.

The above conclusion can be corroborated with a simple statistical analysis of the system in Table 1. For this system, all the anhydride groups on the PA monomers are reacted, and each amine group on a TAPE monomer has a 2/3 chance to be reacted in a fully reacted system. Therefore, the probabilities for a TAPE monomer to react with 0, 1, 2, and 3 PAs are ${\left(\frac{1}{3}\right)}^{3}$, $3\times \frac{2}{3}\times {\left(\frac{1}{3}\right)}^{2}$, $3\times {\left(\frac{2}{3}\right)}^{2}\times \frac{1}{3}$, and ${\left(\frac{2}{3}\right)}^{3}$, respectively. These results are plotted in Figure 4, and they are very close to those from the Flory-Stockmayer theory and the MC simulations using Equation (18). The small differences are due to the fact that the theory and simulations consider a finite system, while the statistical model assumes an infinite system. The comparison thus confirms that the reactions rates based on Equation (18) should be adopted and that the assumption of functional groups of the same type possessing an equal reaction probability made by the Flory-Stockmayer theory is reasonable. From now on, all the data presented in this paper are computed with Equation (18) for the reaction rates. In Section 4.2 and Section 4.3, the focus is on fully reacted stoichiometric systems where $p_{A}=p_{B}=1$. Partially reacted stoichiometric systems where $p_{A}=p_{B}<1$ are discussed in Section 4.4, and nonstoichiometric systems where $p_{A}\neq p_{B}$ are presented in Section 4.5.

### 4.2. Fully Reacted Stoichiometric Systems

For the branched PEIs considered in this paper, type *A* monomers are BPADA and PA, and type *B* monomers are MPD and TAPE, with $f_{1}=1$, $f_{2}=2$, $g_{1}=2$, and $g_{2}=3$. From the Flory theory [8], the gel point is $\alpha_{c}=1/\left(g_{2}-1\right)=1/2$. However, for the systems at hand, the expression of α, which characterizes the level of cross-linking, has to be modified from the original form derived by Flory [8], because each PA monomer as a chain terminator has only one functional group. The modified expression is

(20) $$\begin{array}{ccc} \alpha & = & \sum_{q=0}^{\infty }{\left[p_{A}\left(1-\rho_{1}\right)p_{B}\left(1-\rho_{2}\right)\right]}^{q}p_{A}\left(1-\rho_{1}\right)p_{B}\rho_{2}\\ & = & p_{A}p_{B}\frac{\left(1-\rho_{1}\right)\rho_{2}}{1-p_{A}p_{B}\left(1-\rho_{1}\right)\left(1-\rho_{2}\right)} \end{array}$$

where $\rho_{1}$ is the fraction of functional groups on the terminators (i.e., PA monomers) with respect to all the functional groups on the type *A* monomers, and $\rho_{2}$ is the fraction of functional groups on the branching agents (i.e., TAPE monomers) with respect to all the functional groups on the type *B* monomers. For a fully reacted stoichiometric system, $p_{A}=p_{B}=1$ and Equation (20) can be simplified as

(21) $$\alpha =\frac{\left(1-\rho_{1}\right)\rho_{2}}{\rho_{1}+\rho_{2}-\rho_{1}\rho_{2}}$$

The numbers of monomers can be varied to tune $\rho_{1}$ and $\rho_{2}$, thus putting the fully reacted system below ($\alpha <\alpha_{c}$), around ($\alpha ≃\alpha_{c}$), or beyond ($\alpha >\alpha_{c}$) the gel point. Three such systems are listed in Table 2, where $\rho_{2}$ is changed by varying the numbers of MPD and TAPE monomers. In the MC simulations of these stoichiometric systems, $p_{A}$ and $p_{B}$ are both set to 1, thus allowing the systems to be fully reacted.

**Figure 5:**
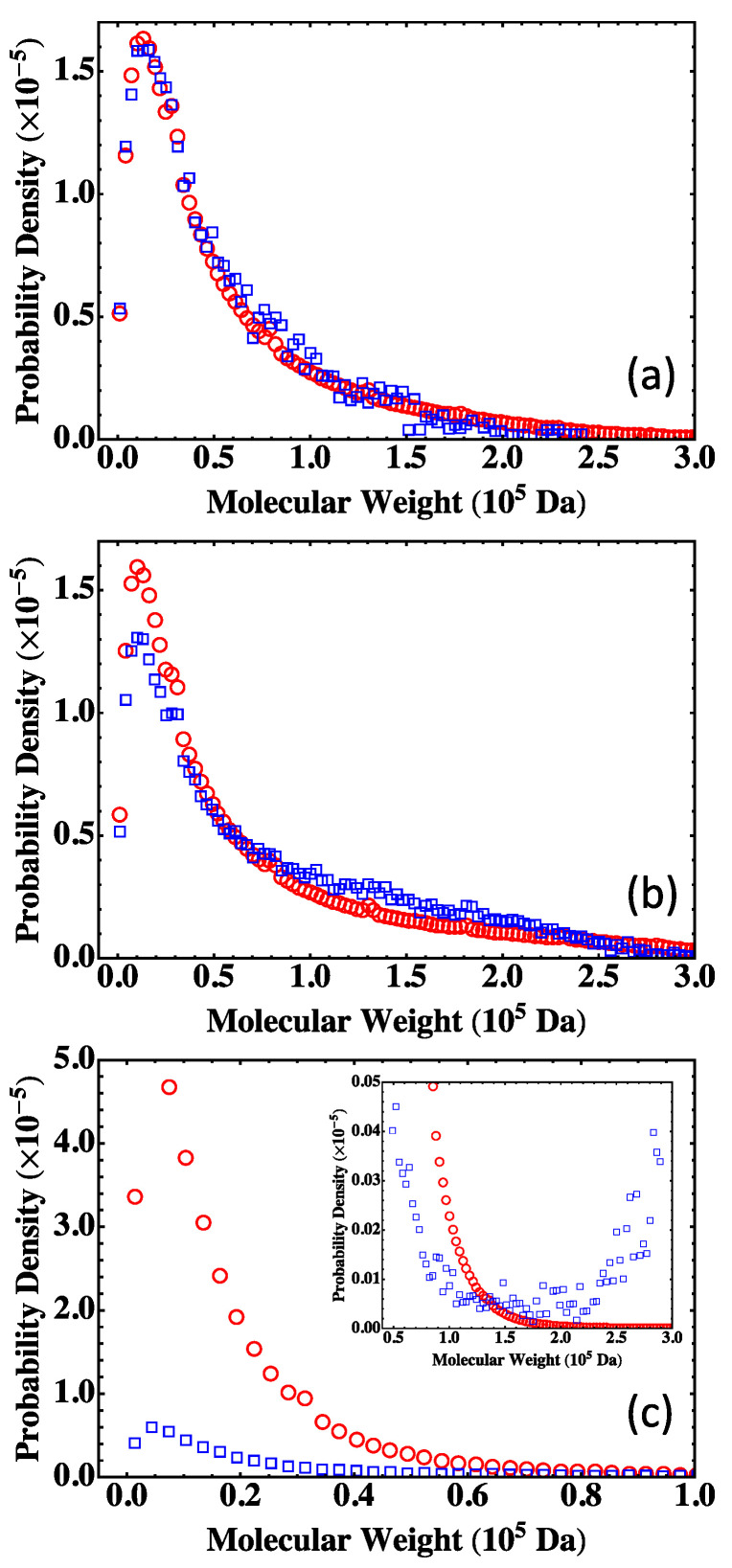
MWD for the three systems in Table 2: (**a**) $S_{<}$ with $\alpha <\alpha_{c}$, (**b**) $S_{≃}$ with $\alpha ≃\alpha_{c}$, and (**c**) $S_{>}$ with $\alpha >\alpha_{c}$, with the inset showing data in the region of high molecular weight. The results are for the Flory-Stockmayer theory (red circles) and the MC simulations (blue squares). The MC results are the averages of 1000 runs for $S_{<}$, and 10,000 runs for $S_{≃}$ and $S_{>}$.

The results on MWD from the Flory-Stockmayer theory and the MC simulations are shown in Figure 5. In this paper, the probability density function of the weight fraction distribution is plotted against molecular weight. The comparison shows that for a system below the gel point such as $S_{<}$ (Figure 5a), the MC results agree well with the Flory-Stockmayer theory. For $S_{≃}$ which is close to the gel point, the Flory-Stockmayer theory overestimates the fraction of low molecular weight polymers and underestimates the fraction of high molecular weight species when compared with the MC results, as shown in Figure 5b.

The discrepancy between the Flory-Stockmayer theory and the MC results becomes more dramatic for systems above the gel point. For $S_{>}$, α=0.743, way above the critical gel point, $\alpha_{c}=0.5$. The Flory-Stockmayer theory predicts a probability density that is about eight times the MC result in the region of low molecular weight, from 0 to about $0.5\times 10^{5}$ Da, as shown in Figure 5c. However, the MC simulations show a significant fraction of polymers in the region of molecular weight that are higher than about $1.5\times 10^{5}$ Da, and these high molecular weight polymers are completely overlooked by the Flory-Stockmayer theory, as shown in the inset of Figure 5c. This discrepancy is not surprising, as beyond the gel point, polymers with a large network structure are expected, and closed loops can frequently emerge in such polymers. The Flory-Stockmayer theory does not consider the formation of loops, and thus cannot accurately predict MWD for systems above the gel point.

### 4.3. Effect of System Size

In experiments, the amount of monomers involved is in the order of moles, i.e., in the order of $10^{23}$. It is thus practically impossible to directly compute MWD from the Stockmayer formula (Equation (2)) for such macroscopic systems. These systems are also out of the reach of MC simulations that typically deal with systems of fewer than $10^{6}$ monomers. A natural question that can be asked is: if the molar ratios are kept unchanged but the numbers of participating monomers are reduced in proportion, can either the Flory-Stockmayer theory or the MC simulations be used to generate an MWD that is applicable to a macroscopic system? To answer this question, four additional systems listed in Table 3 are tested. The smallest system has 10 PA, 134 BPADA, 146 MPD, and 2 TAPE, and is denoted as $S_{1}$. Then, the numbers of monomers are increased ten-fold, fifty-fold, and eighty-fold by keeping the ratios to generate the systems $S_{10}$, $S_{50}$, and $S_{80}$. The subscript of the system label reflects the size ratio with respect to the smallest system, $S_{1}$. In this notation, the system $S_{<}$ in Table 2 is equivalent to $S_{5}$. All these systems are still below the gel point when fully reacted.

**Figure 6:**
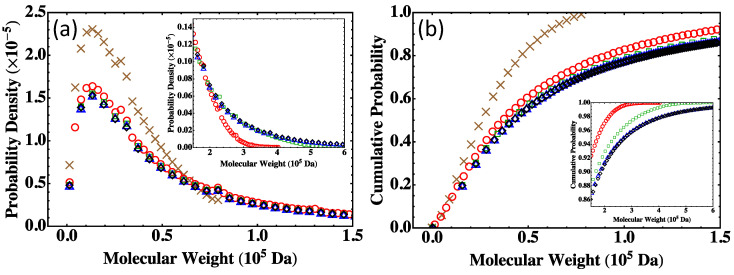
MWD predicted by the Flory-Stockmayer theory for systems with different sizes: (**a**) Probability density and (**b**) cumulative probability. The main panels show the data in the low molecular weight region, while the insets show the data in the high molecular weight region. Data are for $S_{1}$ (brown crosses), $S_{<}$ (red circles), $S_{10}$ (green squares), $S_{50}$ (blue triangles), and $S_{80}$ (black diamonds).

The MWDs predicted by the Flory-Stockmayer theory for $S_{1}$, $S_{<}$, $S_{10}$, $S_{50}$, and $S_{80}$, including the probability density and the cumulative probability, are shown in Figure 6. The main panels are for the region of low molecular weight and the insets show the data in the high molecular weight region. The data show that when the system size is increased, the curves of MWD converge quickly. There is a clear difference between the data for $S_{1}$ and those for $S_{<}$ (i.e., $S_{5}$). However, the difference between $S_{<}$ and $S_{80}$ is very small in the low molecular weight region and only discernible in the tail of the distribution in the region of high molecular weight (see the insets of Figure 6). Furthermore, the results for $S_{50}$ and $S_{80}$ are almost indistinguishable in the entire region of molecular weight relevant to experiments, indicating that these systems are already large enough so that MWD is not affected by the finite system size any more.

**Figure 7:**
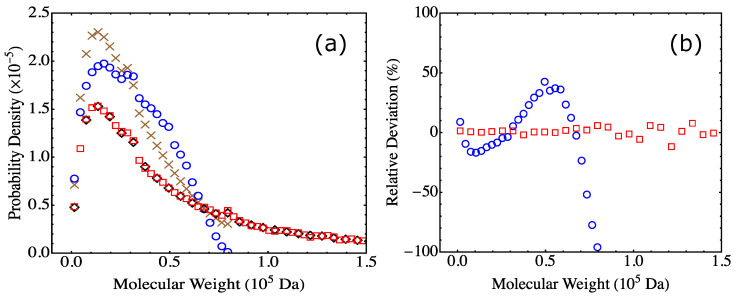
(**a**) MWD with data from for the Flory-Stockmayer theory for $S_{1}$ (brown crosses), the MC simulations for $S_{1}$ (blue circles), the Flory-Stockmayer theory for $S_{80}$ (black diamonds), and the MC simulations for $S_{80}$ (red squares). The MC results are averages of 50,000 runs for $S_{1}$ and 1000 runs for $S_{80}$ (as well as $S_{<}$, $S_{10}$, and $S_{50}$). (**b**) The relative deviation of the MC results from the prediction of the Flory-Stockmayer theory for $S_{1}$ (blue circles) and $S_{80}$ (red squares).

Since $S_{1}$, $S_{<}$, $S_{10}$, $S_{50}$, and $S_{80}$ are all below the gel point, the results on MWD from the Flory-Stockmayer theory and the MC simulations are expected to agree. The comparison between the two is shown in Figure 7a for $S_{1}$ and $S_{80}$ with the relative deviation, defined as ([MC]-[Theory])/[Theory], shown in Figure 7b. For $S_{1}$, some difference is observed between the prediction of the Flory-Stockmayer theory and the MC result because of the small size of this system. An excellent agreement is found between the theory and simulations for $S_{80}$. Similar agreements are also found for $S_{10}$ and $S_{50}$. A good agreement is already discussed earlier for $S_{<}$, as shown in Figure 5a. These comparisons once again confirm that the Flory-Stockmayer theory provides a good description of MWD for systems that are well below the gel point, where ring formation is not a big concern. Below the gel point, both Flory-Stockmayer theory and MC simulations can be applied to a system containing only a few hundred to a few thousand monomers but having the same molar ratios of monomers as a macroscopic system to accurately predict MWD. As discussed earlier, the Flory-Stockmayer theory starts to fail when a system approaches or goes above the gel point. However, in these situations, the MC simulations can still be used to quickly generate an MWD that is applicable to a macroscopic experimental system.

### 4.4. Partially Reacted Stoichiometric Systems

Up to this point, we mainly focus on fully reacted stoichiometric systems as it is possible to compute MWD using the Stockmayer formula, even for a system with a relatively large size such as $S_{80}$. In this and the next sections, we show that the conclusions reached so far also apply to partially reacted and/or nonstoichiometric systems. However, because of the practical difficulty of using the Stockmayer formula to compute MWD when either $p_{A}$ or $p_{B}$, or both, are less than 1, small systems similar to those in Table 2 are used to illustrate the main point.

In this section, partially reacted stoichiometric systems are discussed, where $\sum_{q=1}^{i}f_{q}A_{q}=\sum_{h=1}^{j}g_{h}B_{h}$ but $p_{A}=p_{B}<1$. Five such systems with the same size as $S_{>}$ are listed in Table 4, where the values of $p_{A}$ and $p_{B}$ are increased from 0.95 to 0.99. The corresponding values of α change from about 0.42 to about 0.65, thus enclosing the gelation transition at $\alpha_{c}=0.5$.

**Figure 8:**
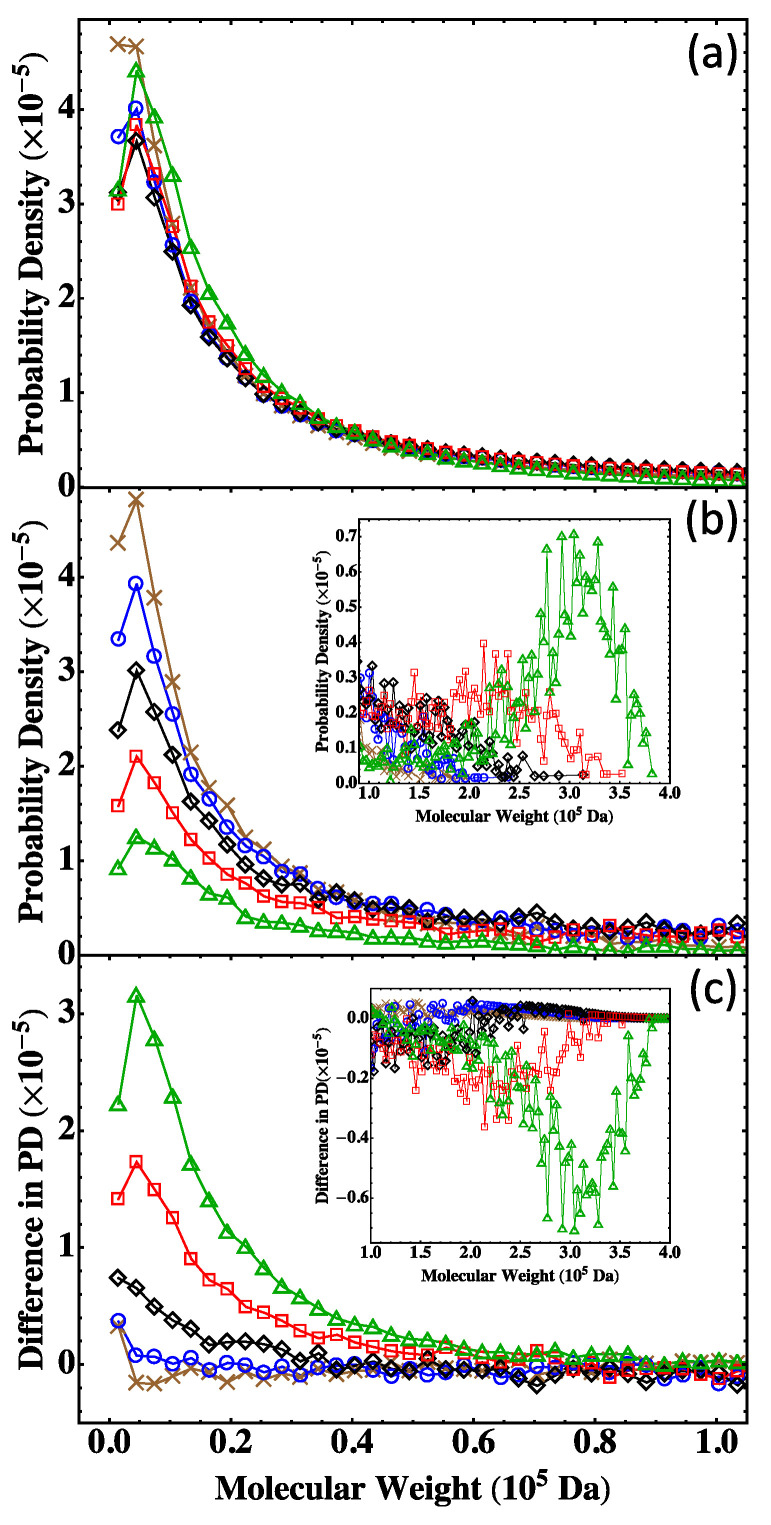
MWD from (**a**) the Flory-Stockmayer theory and (**b**) the MC simulations. The inset of (**b**) shows the MC results in the high molecular weight region. (**c**) Difference in the probability density (PD) from the Flory-Stockmayer theory and the MC simulations, with the inset showing data in the high molecular weight region. The results are for $S^{0.95}$ (brown crosses), $S^{0.96}$ (blue circles), $S^{0.97}$ (black diamonds), $S^{0.98}$ (red squares), and $S^{0.99}$ (green triangles). The MC results are the averages of 1000 runs.

The results on MWD from the Flory-Stockmayer theory and MC simulations at various values of $p_{A}$ and $p_{B}$ are shown in Figure 8a and Figure 8b, respectively. The MWD predicted by the Flory-Stockmayer theory seems to be relatively insensitive to the values of $p_{A}$ and $p_{B}$. However, the MC results show that when the value of $p_{A}$ and $p_{B}$ is increased, the probability density in the low molecular weight region is reduced (see Figure 8b), while that in the high molecular weight region is enhanced (see the inset of Figure 8b). This systematic trend is expected, as when the extent of reaction is increased, more polymers with higher molecular weights are anticipated to form.

To compare the predictions of the Flory-Stockmayer theory to the MC results on MWD, the differences in the probability density are shown for various $p_{A}$ and $p_{B}$ in Figure 8c. It is clear that when $p_{A}$ and $p_{B}$ are small, the systems are below the gel point, and the results from the theory and simulations agree, as for $S^{0.95}$ and $S^{0.96}$. The difference becomes noticeable when the system approaches the gel point, such as $S^{0.97}$. For $S^{0.98}$ and $S^{0.99}$, they are above the gel point, and clear differences in the probability density from the theory and simulations can be noted in both low (see Figure 8c) and high (see the inset of Figure 8c) molecular weight regions. The results for the partially reacted stoichiometric systems thus reaffirm the conclusion that the Flory-Stockmayer theory only applies to systems that are well below the gel point. However, the MC simulations can be used to reliably compute MWD for any systems, no matter if they are below, around, or above the gel point.

### 4.5. Nonstoichiometric Systems

Finally, nonstoichiometric systems are considered, where $\sum_{q=1}^{i}f_{q}A_{q}\neq \sum_{h=1}^{j}g_{h}B_{h}$ and $p_{A}\neq p_{B}$. Three systems with sizes similar to $S_{>}$ are shown in Table 5. The value of $p_{A}$ is fixed at 0.99, but $p_{B}$ varies from 0.93 to 0.97. The numbers of PA, BPADA, and TAPE monomers are all fixed. The number of MPD is varied according to Equation (1). Specifically, when the number of MPD is reduced, the values of $p_{B}$, $\rho_{2}$, and α are all increased. For the three systems in Table 5, $S_{<}^{n}$ is below, $S_{≃}^{n}$ is around, and $S_{>}^{n}$ is above the gel point. Here, the superscript *n* in the system labels indicates that these systems are nonstoichiometric.

**Figure 9:**
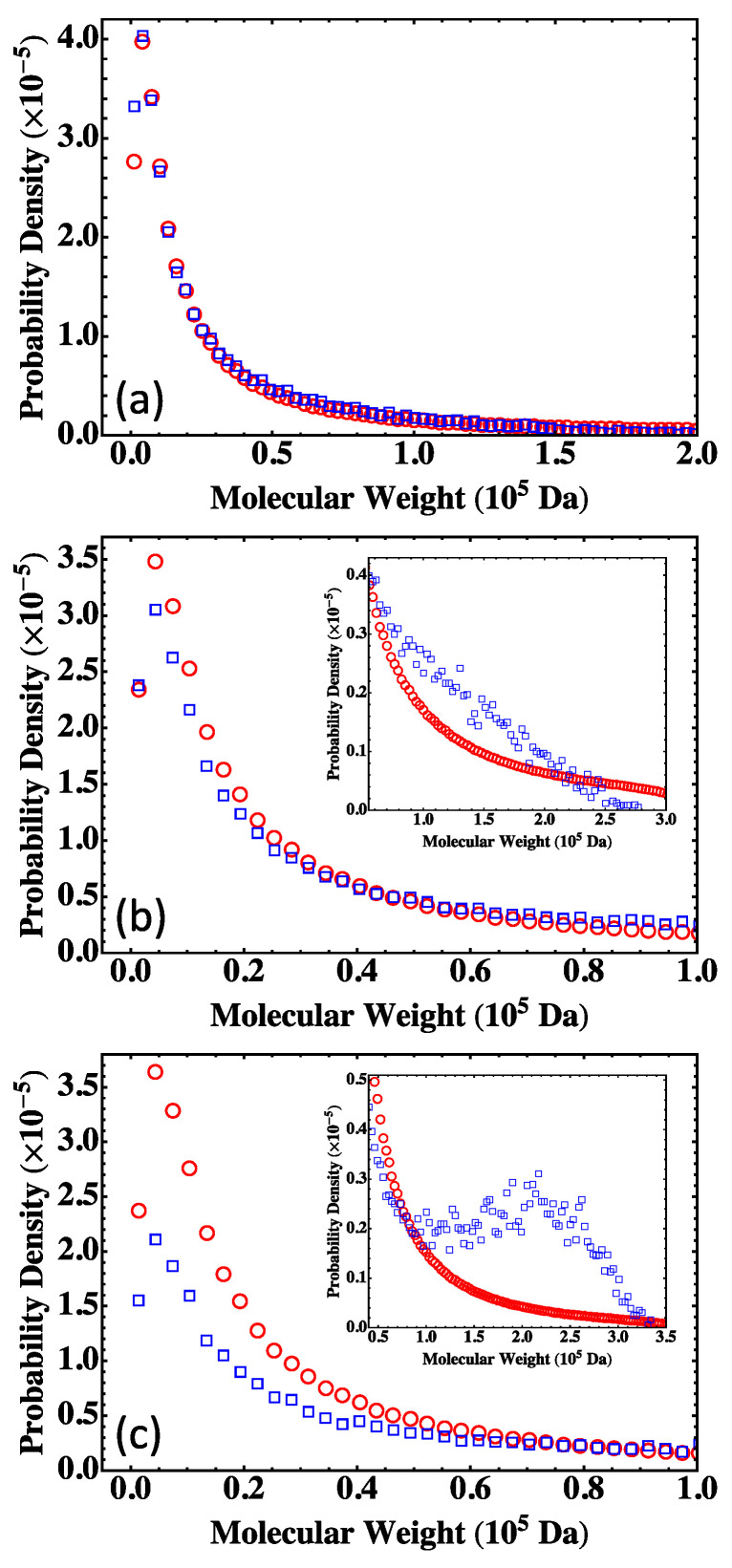
MWD for the three systems in Table 5: (**a**) $S_{<}^{n}$, (**b**) $S_{≃}^{n}$, and (**c**) $S_{>}^{n}$. The results are for the Flory-Stockmayer theory (red circles) and the MC simulations (blue squares). The MC results are the averages of 5000 runs.

The results on MWD for the three nonstoichiometric systems are plotted in Figure 9. For $S_{<}^{n}$ which is below the gel point, the MC results agree with the prediction of the Flory-Stockmayer theory, as shown in Figure 9a. The two start to differ when a system approaches the gel point. An example is shown in Figure 9b for $S_{≃}^{n}$, with α=0.502. For this system, the Flory-Stockmayer theory overestimates the probability of low molecular weight polymers, while it underestimates the probability in the region of molecular weight higher than about $0.5\times 10^{5}$ Da (see the inset of Figure 9b). For $S_{>}^{n}$ which is above the gel point, the MC results on the probability density are smaller than those calculated with the Flory-Stockmayer theory when the molecular weight is lower than about $1\times 10^{5}$ Da (Figure 9c) but higher than the theoretical prediction in the region of high molecular weight (see the inset of Figure 9c). For $S_{>}^{n}$, the MWD has a second peak at around $2\times 10^{5}$ Da, while the Flory-Stockmayer theory predicts a monotonically decaying distribution in this region. The results once more indicate that for systems close to or above the gel point, the MC simulations can properly account for the formation of branched polymers, as rings and loops are properly taken into account, and should be used to generate MWD and to compute the various average molecular weights.

## 5. Conclusions

MC simulations are used to study the formation of branched polymers via a step-growth polymerization mechanism, and the method is applied to the polymerization of the branched PEIs from BPADA (backbone monomer), MPD (backbone monomer), PA (chain terminator), and TAPE (branching agent). All the reactions for this system can be reduced to a condensation reaction between an amine group and a carboxylic anhydride group, and thus, they can be characterized by one reaction rate. The results show that as assumed in the Flory-Stockmayer theory, the reaction rate is determined by the concentrations of the active functional groups on the monomers involved in a specific reaction, and not by the concentrations of the monomers themselves [8,9,10,11,12,13]. A practical approach of using the Flory-Stockmayer theory to compute MWD has been suggested. In particular, the Stockmayer formula on MWD is simplified to a much more tractable form for fully reacted stoichiometric systems. The MC results are then compared to the predictions of the Flory-Stockmayer theory. Both theory and simulations accurately predict MWD for systems well below the gel point that is set by the functionality of the branching agent, though ring formation is not considered by the Flory-Stockmayer theory but is allowed in MC simulations. The agreement between the theory and simulations thus indicates that ring formation is negligible for systems that are well below the gel point. However, for systems close to or above the gel point, the Flory-Stockmayer theory is not applicable, as many cyclic polymers can be produced, and rings and loops can form in highly branched networks. The theory significantly underestimates the fraction of polymers with high molecular weights. For these systems, the MC simulations can still be used to quickly compute MWD that can be used to describe experimental measurements, including the average molecular weights.

The results further indicate that in the MC simulations, a system with only a few hundred to a few thousand monomers, but at the same molar ratios of participating monomers, is large enough to yield converging results on MWD for the region of molecular weight relevant to typical experiments (from 0 to about $3\times 10^{5}$ Da in the case of the branched PEIs). These conclusions have been thoroughly confirmed with simulations for fully reacted, partially reacted, stoichiometric, and nonstoichiometric systems. The MC model presented here is expected to be applicable to a wide range of step-growth polymers.

## Figures and Tables

**Table 1:** System used for checking the way to compute the reaction rates.

Monomer	PA	BPADA	MPD	TAPE
Number	2000	0	0	1000

**Table 2:** Three fully reacted, stoichiometric systems below, around, and beyond the gel point. The first column is the system label. The next four columns list the number of each type of monomers. The values of $\rho_{1}$ and $\rho_{2}$ are determined from the monomer numbers. The value of α is computed using Equation (20). The average molecular weights, $M_{n}$, $M_{w}$, and $M_{z}$, are from the MC simulations.

	PA	BPADA	MPD	TAPE	$\rho_{1}$	$\rho_{2}$	$p_{A}$	$p_{B}$	α	$M_{n}$ (Da)	$M_{w}$ (Da)	$M_{z}$ (Da)
$S_{<}$	50	670	680	10	0.0360	0.0216	1	1	0.366	19,120±33	52,126±545	78,671±2488
$S_{≃}$	50	670	671	16	0.0360	0.0345	1	1	0.481	22,000±15	77,353±307	116,227±499
$S_{>}$	50	670	620	50	0.0360	0.108	1	1	0.743	51,055±125	330,124±458	369,588±348

**Table 3:** Four fully reacted, stoichiometric systems that are all below the gel point, but with the size increased proportionally without changing the molar ratio of monomers. The entries have the same format as in Table 2. The subscript of the system label in the first column indicates the size ratio with respect to the base system, $S_{1}$. The average molecular weights, $M_{n}$, $M_{w}$, and $M_{z}$, are from the MC simulations.

	PA	BPADA	MPD	TAPE	$\rho_{1}$	$\rho_{2}$	$p_{A}$	$p_{B}$	α	$M_{n}$ (Da)	$M_{w}$ (Da)	$M_{z}$ (Da)
$S_{1}$	10	134	136	2	0.0360	0.0216	1	1	0.366	15,742±14	30,829±46	37,334±57
$S_{10}$	100	1340	1360	20	0.0360	0.0216	1	1	0.366	19,799±18	59,940±607	101,321±1441
$S_{50}$	500	6700	6800	100	0.0360	0.0216	1	1	0.366	20,361±4	73,582±619	161,904±2612
$S_{80}$	800	10,720	10,880	160	0.0360	0.0216	1	1	0.366	20,417±3	75,980±550	177,919±2712

**Table 4:** Five partially reacted, stoichiometric systems (i.e., $p_{A}=p_{B}<1$). The entries have the same format as in Table 2. The superscript of the system label indicates the values of $p_{A}$ and $p_{B}$. The first two are below and the other three are beyond the gel point. The average molecular weights, $M_{n}$, $M_{w}$, and $M_{z}$, are from the MC simulations, with 1000 runs for each system.

	PA	BPADA	MPD	TAPE	$\rho_{1}$	$\rho_{2}$	$p_{A}$	$p_{B}$	α	$M_{n}$ (Da)	$M_{w}$ (Da)	$M_{z}$ (Da)
$S^{0.95}$	50	670	620	50	0.0360	0.108	0.95	0.95	0.419	5965±3	24,779±331	47,952±824
$S^{0.96}$	50	670	620	50	0.0360	0.108	0.96	0.96	0.462	7386±5	36,488±488	69,368±1097
$S^{0.97}$	50	670	620	50	0.0360	0.108	0.97	0.97	0.513	9655±10	58,237±807	103,512±1504
$S^{0.98}$	50	670	620	50	0.0360	0.108	0.98	0.98	0.574	13,780±23	105,576±1435	165,690±2080
$S^{0.99}$	50	670	620	50	0.0360	0.108	0.99	0.99	0.649	22,710±70	200,514±1915	266,624±2083

**Table 5:** Three partially reacted, nonstoichiometric systems (i.e., $p_{A}\neq p_{B}$, and both are less than 1) below, around, and beyond the gel point. The entries have the same format as in Table 2. The average molecular weights, $M_{n}$, $M_{w}$, and $M_{z}$, are from the MC simulations, with 5000 runs for each system.

	PA	BPADA	MPD	TAPE	$\rho_{1}$	$\rho_{2}$	$p_{A}$	$p_{B}$	α	$M_{n}$ (Da)	$M_{w}$ (Da)	$M_{z}$ (Da)
$S_{<}^{n}$	50	670	664	50	0.0360	0.101	0.99	0.93	0.445	7225±2	33,130±195	62,388±448
$S_{≃}^{n}$	50	670	649	50	0.0360	0.104	0.99	0.95	0.502	9530±4	54,269±349	96,424±656
$S_{>}^{n}$	50	670	634	50	0.0360	0.106	0.99	0.97	0.569	13,824±10	103,074±624	162,837±915

## Data Availability

The data presented in this study are available on request from the corresponding author. The data are not publicly available due to the large size.

## Abbreviations

The following abbreviations are used in this manuscript:

MC	Monte Carlo
MWD	molecular weight distribution
PEI	polyetherimide
BPADA	4,4${}^{\prime }$-bisphenol A dianhydride
MPD	m-phenylenediamine
PA	phthalic anhydride
TAPE	tris[4-(4-aminophenoxy)phenyl] ethane
PD	probability density
$M_{n}$	number-average molecular weight
$M_{w}$	weight-average molecular weight
$M_{z}$	*z*-average molecular weight
